# Μaximal Fat Oxidation During Cycle Ergometer Protocols in Obese Adults: A Scoping Review

**DOI:** 10.3390/diseases14010004

**Published:** 2025-12-24

**Authors:** Konstantinos Anagnostopoulos, Apostolos Spassis, Christos Kokkotis, Ilias Smilios, Athanasios Chatzinikolaou, Helen T. Douda, Alexios Batrakoulis

**Affiliations:** 1 Department of Physical Education and Sport Science, School of Physical Education, Sport Science and Occupational Therapy, Democritus University of Thrace, 69100 Komotini, Greece; aspassis@phyed.duth.gr (A.S.); ckokkoti@affil.duth.gr (C.K.); ismilios@phyed.duth.gr (I.S.); achatzin@phyed.duth.gr (A.C.); edouda@phyed.duth.gr (H.T.D.); 2 Department of Physical Education and Sport Science, School of Physical Education, Sport Science and Dietetics, University of Thessaly, 42100 Trikala, Greece

**Keywords:** Fatmax, obesity, overweight, metabolic flexibility, exercise testing protocols, metabolic health

## Abstract

Maximal fat oxidation (MFO) rate and the intensity at which it occurs (Fatmax) are key indicators of metabolic flexibility, yet their assessment in obese populations poses methodological challenges. This scoping review synthesizes evidence from 23 studies investigating protocols for determining Fatmax and MFO during cycle ergometry. Across studies, obese and sedentary participants followed testing procedures, typically involving lower initial workloads, smaller workload increments, and longer stage durations than those used for fitter individuals. In obese populations, Fatmax generally occurred at 30–50% of VO_2_ peak, compared with values exceeding 60% in trained participants. While the reliability of Fatmax was acceptable, greater variability was observed for MFO rate. Fitness level appeared to exert a stronger influence than adiposity on fat oxidation, with obesity often associated with a left-shifted fat oxidation curve. Additional factors such as gender, developmental stage, insulin resistance, and type 2 diabetes further modulated these responses. Importantly, short-term training interventions, including moderate-intensity exercise, high-intensity interval training, and Fatmax-targeted protocols, consistently enhanced MFO and shifted Fatmax toward higher intensities, with favorable effects on insulin sensitivity and metabolic health. In contrast, nutritional and supplementation studies provided limited evidence of additional benefits. Overall, Fatmax assessment is feasible in obese populations when appropriate methodological adjustments are applied, and exercise interventions can rapidly enhance fat oxidation capacity. Future research should focus on protocol standardization, mechanistic exploration, and long-term interventions to clarify the role of Fatmax in obesity management and its potential clinical applications.

## 1. Introduction

Obesity represents a major and rapidly growing public health concern, characterized by excessive adipose tissue accumulation, chronic low-grade systemic inflammation, and reduced metabolic adaptability [1]. The prevalence and severity of obesity among adolescents, along with its associated health complications, continue to rise across industrialized nations. Extensive research has established obesity as a major risk factor for various cardio-metabolic disorders, including insulin resistance, type 2 diabetes, hypertension, coronary heart disease, and obstructive sleep apnea. Physical activity is widely recognized as an effective strategy to mitigate these risks and is recommended as an adjunct to calorie-restricted dietary interventions for the management of obesity [2]. Regular physical activity and structured exercise training have consistently been shown to enhance fat oxidation in both healthy and obese individuals. Therefore, a deeper understanding of the factors that influence fat oxidation rates at rest and during exercise is of crucial importance [3].

MFO, defined as the highest absolute rate of lipid oxidation, is typically expressed in g/min, and the exercise intensity at which MFO occurs (Fatmax Stanley), defined as the exercise intensity at which MFO is achieved, is typically expressed as a percentage of maximal oxygen uptake (%VO_2_ max). These are recognized biomarkers of an individual’s capacity to oxidize lipids during exercise. They are associated with both metabolic health and endurance performance [4,5,6,7]. It is well established that fat oxidation increases with rising exercise intensity up to a certain point (commonly around 50–65% of VO_2_max in many trained individuals), after which it declines at higher intensities [4,8]. Maunder et al. [4] describe this relationship as a “parabolic” curve, where lower intensities elicit reduced lower fat oxidation, moderate intensities maximize fat oxidation, and higher intensities lead metabolism towards predominantly carbohydrate utilization. Determinants of MFO and Fatmax include training status, substrate availability (e.g., carbohydrate vs. lipid), sex and body composition, exercise modality, timing and nutritional state, as well as mitochondrial and transport capacity for fatty acid oxidation [4,9]. Furthermore, recent findings highlight the importance of enhanced lipolytic activation and subsequent muscle-level fatty acid oxidation as key factors regulating Fatmax intensity. Enzymatic activity related to triacylglycerol lipase function and the availability of circulating free fatty acids have been shown to critically influence lipid utilization during exercise [10,11,12].

During incremental exercise, the contribution of lipids to energy production is regulated by substrate availability, hormonal balance, and mitochondrial transport capacity [4]. At low exercise intensities, catecholamine-stimulated lipolysis increases plasma free fatty acids (FFA), whereas insulin exerts an antilipolytic effect, together shaping the availability of FFA to skeletal muscle [13]. As exercise intensity rises, carbohydrate oxidation increasingly dominates due to enhanced glycolytic flux and the accumulation of malonyl-CoA, which inhibits carnitine palmitoyltransferase-1 (CPT-1) and restricts mitochondrial fatty-acid entry [9,14]. Concurrently, whole-body fat oxidation declines at high intensities as a result of reduced fatty-acid delivery and uptake, as well as constraints on mitochondrial transport, giving rise to the characteristic “parabolic” fat oxidation curve across intensities [4,8].

In individuals with obesity, alterations in lipolysis, fatty acid delivery to skeletal muscle, insulin and catecholamine responses, as well as impairments in mitochondrial transport and oxidation, may shift the Fatmax intensity or reduce MFO due to specific metabolic restrictions. These metabolic alterations, such as insulin resistance and reduced capacity for FFA delivery, necessitate specific methodological adaptions when assessing MFO in this population, providing the biological rationale for the focus of this review [15,16]. Strategies aiming to improve fat metabolism may help attenuate the adverse effects of obesity and yield meaningful clinical benefits. This perspective aligns with the exercise guidelines for individuals with obesity issued by the American College of Sports Medicine and the World Health Organization, which emphasize increasing energy expenditure to achieve a negative energy balance and promote weight management through exercise [1,17]. However, emerging evidence suggests that focusing on enhancing fat oxidation during exercise, rather than merely increasing total energy expenditure, may represent a more targeted and effective strategy for the prevention and treatment of obesity [1,17,18]. Therefore, the development of regular exercise programs specifically designed to maximize fat oxidation and reduce adiposity is of paramount importance [2].

Over the past two decades, a substantial body of evidence has demonstrated that exercising at Fatmax can improve body composition, cardiorespiratory fitness (CRF), endocrine function, and metabolic flexibility in individuals with obesity and type 2 diabetes [1,17,19]. It is important to note that MFO declines with age in healthy individuals [20]. Moreover, MFO rates are typically higher during weight-bearing activities such as running and rowing likely due to the recruitment of a greater muscle mass, performed at Fatmax compared to stationary cycling [1,4]. Therefore, training recommendations designed to optimize the MFO rate should consider both the individual’s age and the mode of exercise performed [1]. To assess fat oxidation across a wide range of exercise intensities, researchers have developed and validated a time-efficient protocol that allows all measurements to be completed with a single laboratory session. Commonly referred to as the Fatmax test, this approach determines both the MFO rate and Fatmax, typically expressed as a percentage of maximal oxygen uptake (%VO_2_max) [4,5].

Initially developed for use on a cycle ergometer, the protocol involves continuous, incremental increases in workload—by 35 W every three minutes—until volitional exhaustion [21]. Throughout the test, breath-by-breath gas exchange is continuously measured, and fat oxidation rates are calculated for each stage using established stoichiometric equations [21]. Following its initial application, a treadmill-based version of the Fatmax test was also introduced [22]. Reproducibility studies have since reported minimal intra-individual variability in MFO and Fatmax values obtained using this method [22]. However, the graded exercise protocol developed by Achten et al. was not originally designed for individuals with obesity or those with low aerobic capacity. As a result, such participants often complete only two to three stages of the original test, making precise determination of Fatmax challenging. To address this limitation, several modified graded exercise protocols, starting at lower workloads (30–50 W), have been implemented to assess Fatmax in individuals with obesity [23,24,25,26]. Nonetheless, to date, a consensus on a universally validated, standardized obesity-specific protocol for determining MFO during cycle ergometry remains lacking [24].

Most studies indicate that, in individuals with obesity, Fatmax typically occurs between 40% and 50% of VO_2_max [1,4,17]. However, substantial inter-individual variability has been reported, ranging from 30% to 65% of VO_2_max, independent of gender. This variability appears to be influenced by factors such as CRF, body composition, and genetic differences, implying that at a given moderate exercise intensity, some individuals may be exercising within their Fatmax zone while others are operating below or above it. For that reason, fat oxidation rates vary substantially among individuals. Therefore, moderate-intensity exercise should not be assumed to equate to Fatmax training, as achieving MFO requires individualized assessment and exercise prescription [17].

Despite increasing interest in Fatmax testing, much of the existing evidence derives from normal-weight or athletic populations, often using protocols that may not be appropriate for individuals with obesity. Considerable heterogeneity in testing procedures, including differences in workload increments, stage duration, and starting stage/intensity, further limits comparability between studies and hampers the establishment of clear, evidence-based guidelines. Moreover, although several protocols for determining MFO/Fatmax during cycle ergometry have been proposed and applied over the past two decades, no universally accepted, obesity-specific protocol for determining MFO during cycle ergometry has been validated against continuous exercise performed at multiple intensities. A comprehensive synthesis of the available literature is needed to identify existing approaches, highlight methodological gaps, and guide both future research and practical application. Moreover, the current state of the literature lacks a critical synthesis of these methodological gaps and their impact on MFO accuracy. A comprehensive synthesis of the available literature is needed to identify existing approaches, highlight methodological gaps, and propose a pathway towards standardization to guide both future research and practical application. This scoping review aims to systematically map and summarize the protocols that have been employed to determine MFO during cycle ergometry in individuals with obesity and overweight.

## 2. Materials and Methods

This study was registered on the Open Science Framework [available at https://doi.org/10.17605/OSF.IO/N5V6G (accessed on 13 August 2025)]. The scoping review was conducted in accordance with the 22-item PRISMA-ScR checklist to ensure a comprehensive, consistent, and transparent review process (Table S1) [27,28]. Adhering to the PRISMA-ScR framework was intended to uphold rigorous methodological standards, enhance transparent reporting, and strengthen the reliability and validity of the qualitative synthesis.

### 2.1. Literature Searches

A systematic literature search was carried out in the PubMed and Scopus databases on 5 June 2025. This combined approach was intended to ensure the inclusion of all pertinent studies. The search strategy utilized the following keywords and phrases, with the Title/Abstract filter applied in each database and covered the period from 1 January 2003 to 5 June 2025. The following keywords were used: “maximal fat oxidation” OR “peak fat oxidation” OR “maximal lipid power” AND FATmax OR LIPOXmax AND obese OR obesity AND “cycle ergometer” OR cycle OR cycling. To address potential literature gaps and ensure a comprehensive mapping of all foundational protocols, particularly those related to LIPOXmax, a supplementary targeted search was also conducted within the *Science & Sports* (via ScienceDirect) journal.

### 2.2. Eligibility Criteria

#### 2.2.1. Inclusion Criteria

Only peer-reviewed journal articles were included to ensure quality and reliability. The review encompassed studies from 1 January 2003 to 5 June 2025, focusing specifically on original quantitative research that employed a cycle ergometer protocol to assess Fatmax and MFO in individuals with obesity or overweight, regardless of the study design (e.g., acute trials, cross-sectional studies, or chronic intervention studies).

#### 2.2.2. Exclusion Criteria

To capture studies reflecting the evolution of Fatmax and MFO assessment protocols on cycle ergometers, only articles published from 2002 onwards were considered. This time frame was selected because the first validated Fatmax protocol was introduced in the early 2000s, and subsequent research has since refined and adapted these protocols, particularly for obese populations [21]. Studies were excluded if they were conference proceedings, books, non-English publications, unrelated to exercise modality (cycle ergometer), did not assess MFO or Fatmax, or provided insufficient information to determine the protocol used. Review articles and studies with inaccessible full texts were also omitted to ensure thorough analysis and avoid redundancy.

### 2.3. Data Extraction

The study selection was conducted independently by two reviewers (K.A. and C.K.) in three stages: duplicate removal, title/abstract screening, and full-text review. Any disagreements were resolved through discussion. Data extraction was also performed independently by both reviewers using a standardized, piloted charting form to ensure consistency. Studies meeting predefined criteria, including sample size, age (years), body mass index (BMI) (kg/m^2^), GXT (Cycle Ergometer), fasting condition, VO_2_max (mL/kg/min), MFO (g/min), Fatmax (%VO_2_peak) and fitness status, were included in the qualitative synthesis. A descriptive synthesis summarized the charted data, ensuring the final selection was both relevant and methodologically robust.

## 3. Results

**Figure 1:**
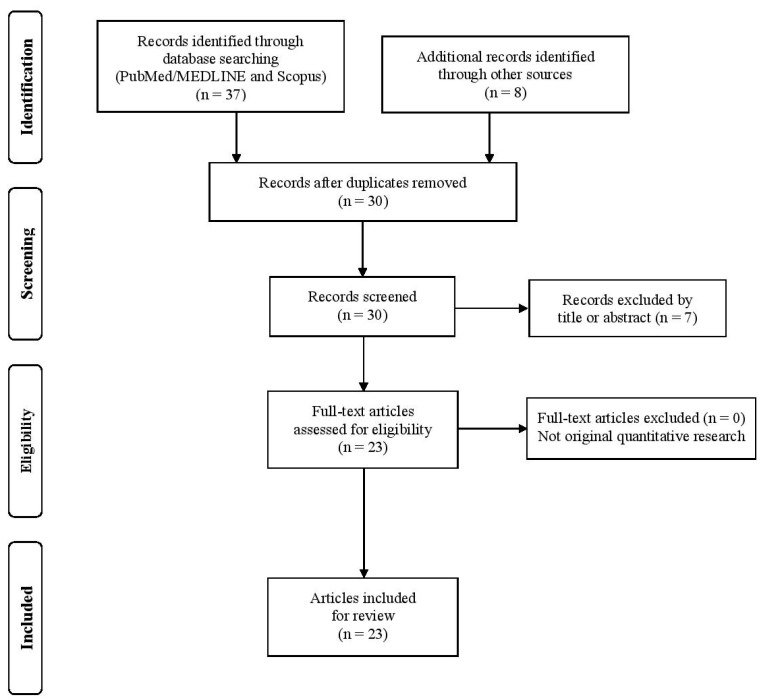
Flow diagram of screening methodology.

The initial search yielded 28 articles after duplicate removal. Following title and abstract screening and a targeted supplementary search, a total of 23 studies met the inclusion criteria and were included in the qualitative synthesis. The study selection process is illustrated in Figure 1, in accordance with PRISMA-ScR guidelines.

These studies investigated protocols for assessing MFO and Fatmax intensity during cycle ergometry in individuals with obesity and overweight, addressing methodological issues, the influence of fitness and body composition influences, sex differences, pediatric and clinical cohorts, as well as both exercise- and nutrition-based interventions (Table 1).

### 3.1. Protocol Characteristics

Protocols used for sedentary and obese participants generally began at very low workloads (20–35 W) and employed small incremental steps (15–17.5 W). Stage durations were typically extended (4–6 min) to allow VO_2_ and VCO_2_ to stabilize, in contrast to the shorter ≈3 min stages used in fitter individuals. This methodological adaptation, observed across the majority of included studies, is essential because longer stage durations (typically 4–6 min) are needed to allow VO_2_ and VCO_2_ to stabilize and minimize the risk of RER drift, which can lead to the underestimation of Fatmax and MFO values. In some cases, discontinuous graded tests were employed, with 3–4 min of rest between stages to avoid premature fatigue. Testing intensities were often limited to low-to-moderate ranges (20–60% VO_2_peak), reflecting the observation that Fatmax in sedentary and obese individuals occurs at ~30–50% VO_2_peak, whereas in trained populations, values frequently exceeded 60%. Most studies also required strict fasting (≥10–12 h) to minimize the influence of recent nutritional intake on substrate oxidation.

Across the included studies, indirect calorimetry during continuous graded cycling was the most commonly used approach for determining Fatmax and MFO. Several studies also examined validation and reliability. For example, Dandanell et al. (2017) compared a graded protocol with a short continuous exercise (SCE) protocol in participants with obesity, reporting strong inter-method reliability (ICC ≈ 0.72–0.75) but substantial intra-individual variability, with Fatmax observed around 42–45% VO_2_ max [24]. Similarly, Chrzanowski et al. (2020) demonstrated acceptable day-to-day reliability for Fatmax, while variability remained higher for MFO [25]. The observed variability in reliability outcomes (higher consistency for Fatmax intensity vs. greater variability for MFO rate) highlights a core methodological weakness across the literature, necessitating cautious interpretation when applying MFO for individualized prescription. Additional evidence further supports the use of prolonged steady-state stages for accurate determination of Fatmax/LIPOXmax in overweight and obese populations [39]. Specifically, protocols employing five consecutive 6 min workloads corresponding to approximately 20–60% of maximal power output, performed under overnight fasting conditions and assessed via indirect calorimetry during cycle ergometry, demonstrated stable substrate oxidation profiles suitable for identifying maximal lipid oxidation. In line with these methodological considerations, prolonged steady-state protocols using 5–6 min cycling stages under fasting conditions have been systematically applied in obese populations to ensure stabilization of gas exchange variables and accurate determination of maximal lipid oxidation [39,46].

### 3.2. Influence of Fitness, Obesity, and Mechanistic Insights

A consistent finding across studies is the modifying effect of obesity and fitness status on fat oxidation. Adults with obesity often exhibit a left-shifted and narrower fat-oxidation curve, characterized by reduced reliance on fat at higher exercise intensities despite elevated circulating non-esterified fatty acids (NEFA). For instance, Lanzi et al. (2014) [42] reported that obese participants achieved a MFO rate of approximately 0.28 ± 0.08 g·min^−1^ occurring at ~47 ± 3% VO_2_peak, which compares unfavorably with lean individuals who reached about 0.40 ± 0.09 g·min^−1^ at ~55 ± 4% VO_2_peak. This confirms that fat oxidation peaks at lower relative intensities in obesity.

However, the primary determinant appears to be cardiorespiratory fitness (CRF) rather than adiposity itself. Croci et al. (2014) [37] reported that once fitness level was accounted for, obesity did not independently reduce MFO. In their study, obese (“HiFat”) and lean (“LoFat”) individuals with similar fitness levels exhibited comparable Fatmax values (~45% VO_2_max) and similar MFO rates (0.42 ± 0.16 vs. 0.38 ± 0.19 g·min^−1^, respectively), supporting the notion that CRF is the dominant determinant of overall lipid oxidation capacity. Similarly, Ara et al. (2011) [31] observed preserved mitochondrial function and normal oxidative enzyme activity in both the arm and leg muscles of obese adults, despite differences in body composition. Obese participants exhibited an MFO (0.40 ± 0.04 g·min^−1^) and Fatmax (~47.4 ± 1.5% VO_2_max) comparable to post-obese and lean counterparts.

These findings suggest that whole-body differences in fat oxidation capacity likely reflect systemic limitations rather than intrinsic mitochondrial impairment at the muscle level, including:

Impaired Substrate Delivery: Alterations in lipolysis, resulting from elevated basal insulin concentrations and blunted catecholamine responses, suppress the release of Free Fatty Acids (FFA) from adipose tissue. This limits the availability of circulating FFA to skeletal muscle.

Hormonal and Regulatory Constraints: As exercise intensity rises, enhanced glycolytic flux leads to the accumulation of malonyl-CoA, which acts as a potent inhibitor of Carnitine Palmitoyltransferase-1 (CPT-1). CPT-1 is the rate-limiting enzyme for the transport of long-chain fatty acids into the mitochondria for β-oxidation. This mechanism causes the characteristic decline in fat oxidation at higher intensities.

Metabolic Status: Furthermore, metabolic health status (e.g., insulin resistance and type 2 diabetes) independently modulates these responses, with insulin-resistant individuals often displaying reduced MFO even when matched for BMI.

In summary, the reduced and left-shifted fat oxidation curve observed in obesity stems primarily from a complex interplay between low CRF and systemic metabolic dysfunction, including limitations in substrate delivery and hormonal signaling, rather than a failure of the muscle’s inherent oxidative machinery.

### 3.3. Pediatric and Clinical Populations

In youth, Fatmax occurred at lower relative intensities in obese children and adolescents compared with their lean peers, with values declining further across pubertal progression [47]. Lazzer et al. (2007) reported that severely obese adolescents exhibited Fatmax of approximately ≈41 ± 3% VO_2_max (≈58% HRmax), with higher absolute fat oxidation in boys than in girls (MFO: boys 0.32 ± 0.02 vs. girls 0.25 ± 0.02 g·min^−1^) [2]. Consistently, Zunquin et al. (2009) demonstrated in obese pubertal boys a progressive decline in fat oxidation across puberty (Pre-pubertal: Fatmax 49.5 ± 1.6% VO_2_max; MFO 0.44 g·min^−1^ → Pubertal: Fatmax 47.4 ± 1.3%; MFO 0.41 g·min^−1^ → Post-pubertal: Fatmax 45.0 ± 0.9%; MFO 0.34 g·min^−1^) [47]. Despite these differences, when obese and non-obese adolescents present similarly low cardiorespiratory fitness (CRF), fat oxidation at equivalent relative workloads is broadly comparable. Additionally, Makni et al. (2012) showed that a 6 min walk test can predict MFO in obese children (n = 131), providing a practical proxy for laboratory assessment [43].

Clinical comorbidities also modulate fat oxidation. In sedentary women with overweight/obesity and insulin resistance, Cancino-Ramírez et al. (2018) observed lower MFO despite similar Fatmax values, and reported that higher VO_2_max and MFO were associated with lower insulin resistance [34]. In individuals with type 2 diabetes, Mogensen et al. (2009) found generally normal MFO (T2D 0.28 ± 0.02 vs. controls 0.32 ± 0.03 g·min^−1^) with appropriate increases following aerobic training; Fatmax values were ~39.7 ± 3.9% vs. 43.7 ± 4.1% VO_2_max (T2D vs. controls) [44]. In pediatric studies, test design critically affected outcomes. Crisp et al. (2012) [36] demonstrated in overweight boys that stage duration alters measured oxidation and Fatmax estimation accuracy. MFO was 0.303 ± 0.08 g·min^−1^ with graded 3 min stages versus 0.328 ± 0.06 g·min^−1^ with prolonged constant-load stages, while Fatmax remained approximately 53 ± 10% VO_2_peak in both approaches [36].

### 3.4. Sex Differences

Sex-related differences in fat oxidation have also been reported. Men generally achieve higher absolute MFO than women, typically ~0.40–0.45 g·min^−1^ vs. ~0.30–0.35 g·min^−1^, respectively, while Fatmax, expressed as %VO_2_peak, is broadly similar (~50–60%) across sexes [6,48]. In obese adults, Haufe et al. (2010) reported MFO = 0.30 ± 0.02 g·min^−1^ in men versus 0.23 ± 0.01 g·min^−1^ in women (*p* < 0.05), with the Fatmax being approximately 42 ± 2.2% of VO_2_max in men and 43 ± 1.7% in women, indicating lower absolute MFO in obese women despite similar relative Fatmax [26]. Similarly, in healthy cohorts, Chenevière et al. (2011) showed higher peak whole-body fat oxidation in men (~0.60 ± 0.12 g·min^−1^ at ~60% VO_2_max) than in women (~0.42 ± 0.10 g·min^−1^ at ~55% VO_2_max), suggesting that sex differences in absolute lipid oxidation primarily reflect differences in lean mass and oxidative capacity rather than distinct substrate preference [48]. Overall, these observations align with Venables et al. (2005), who identified sex, body composition, and fitness as key determinants of inter-individual variability in fat oxidation, while Fatmax (%VO_2_peak) remains relatively comparable between sexes [6].

### 3.5. Exercise Interventions

Interventional studies highlighted the plasticity of fat oxidation. Short-term training (2–8 weeks) consistently increased MFO and shifted Fatmax toward higher intensities, even in sedentary individuals with obesity. In men with obesity, both moderate-intensity and high-intensity interval training (HIIT) improved fat oxidation capacity, with HIIT yielding greater reductions in perceived exertion [30]. Lanzi et al. (2015) observed comparable aerobic and metabolic fitness gains from HIIT and Fatmax-targeted training in men with Class II–III obesity, with the Fatmax-focused program producing superior improvements in insulin resistance [41].

In women with overweight/obesity, Besnier et al. (2015) reported that individualized Fatmax-based training combined with a fruit- and vegetable-rich diet yielded greater fat mass reduction and metabolic improvement than diet alone [32]. In individuals with metabolic syndrome or diabetes, low-intensity endurance exercise prescribed at Fatmax enhanced body composition and insulin sensitivity [44]. Weight-loss interventions generally increased MFO, particularly when normalized to fat-free mass [15]. In sedentary Chinese adults with obesity, a combined exercise program improved both body composition and fat oxidation outcomes [35].

Finally, in men with obesity undergoing lifestyle modification, Ipavec-Levasseur et al. (2015) [40] demonstrated a substantial increase in whole-body MFO. However, a one-hour cycling session at Fatmax did not deplete intramyocellular lipid stores in the soleus either before or after the intervention, suggesting that plasma free fatty acids were the primary substrate utilized.

Long-term interventions based on individualized LIPOXmax prescription have also been reported. In a one-year real-life follow-up study, endurance exercise targeted at maximal lipid oxidation resulted in clinically meaningful weight loss and improved adherence in adults with overweight and obesity, despite the absence of a restrictive diet [39].

### 3.6. Nutritional and Supplementation Findings

Nutritional interventions investigated fat oxidation remain limited. In a randomized crossover trial, dihydrocapsiate supplementation did not enhance energy expenditure or fat oxidation during aerobic cycling in men with overweight and obesity [45].

## 4. Discussion

This scoping review synthesized evidence from 23 studies assessing the MFO rate and the corresponding Fatmax during cycle ergometry in individuals with overweight and obesity. Overall, the findings highlight the methodological adaptations needed for these populations, the substantial influence of fitness status and metabolic health on fat oxidation, and the potential of cycling interventions to enhance both Fatmax and MFO.

### 4.1. Methodological Considerations

Protocols designed for individuals with overweight and obesity consistently differed from those used in lean/normal weight or trained populations. The need for lower starting workloads, smaller increments, and longer stage durations reflects the physiological constraints of sedentary or metabolically impaired participants [24]. This approach aligns with earlier methodological work emphasizing that failure to allow sufficient steady-state conditions can underestimate Fatmax and MFO [24]. Notably, the observed intra-individual variability highlights the limitations of Fatmax as a precise prescription tool, echoing previous debates about its reproducibility in athletic cohorts [25]. Nevertheless, the relatively higher consistency of Fatmax compared with MFO suggests its continued value as a population-level marker of substrate use, while caution should be exercised when applying it for individualized training guidance [25]. Collectively, these methodological discrepancies underline those differences in stage duration, workload increments, and protocol design are not trivial, but can substantially alter both the magnitude and the apparent intensity of MFO, thereby contributing to the wide heterogeneity observed across studies.

### 4.2. Fitness, Obesity and Metabolic Health

An important insight from this review is that cardiorespiratory fitness appears to outweigh adiposity per se in determining Fatmax. Although individuals with obesity often exhibit a left-shifted fat oxidation curve, comparative studies adjusting for fitness demonstrated that the primary determinant is cardiorespiratory capacity [37]. This finding aligns with broader exercise physiology literature demonstrating that endurance training induces muscle adaptations, such as increased mitochondrial density, enhanced enzyme activity, and improved lipid transport, that collectively contribute to greater MFO. Furthermore, the preserved muscle-level oxidative function observed in obese adults further reinforces the notion that systemic factors, including substrate delivery, hormonal regulation, and insulin sensitivity, rather than intrinsic mitochondrial impairment, may limit whole-body fat oxidation [31].

In clinical and pediatric populations, distinct patterns of fat oxidation have emerged. Youth with obesity consistently demonstrate lower Fatmax relative to VO_2_peak, with further reductions observed across pubertal progression [47]. These changes likely reflect both developmental hormonal changes and lower habitual physical activity. Such observations are consistent with longitudinal data indicating a decline in metabolic flexibility during adolescence, particularly in the presence of obesity. Sex differences were less consistent, although men generally exhibit higher absolute MFO, relative Fatmax remains comparable across sexes, suggesting that differences are primarily attributable to body size, muscle mass, and hormonal profile rather than intrinsic substrate utilization [42].

Metabolic health also emerged as an independent determinant of fat oxidation. Insulin-resistant individuals, even when matched for BMI, displayed impaired MFO, consistent with mechanistic evidence linking insulin resistance to reduced lipolysis and impaired fatty acid transport [34]. Interestingly, individuals with type 2 diabetes are capable of achieving normal fat oxidation responses following aerobic training, suggesting that exercise can partially restore metabolic flexibility even in the context of advanced metabolic disease [44].

From a mechanistic perspective, Fatmax and MFO reflect the dynamic regulation of lipid oxidation by hormonal and mitochondrial factors. At moderate exercise intensities, catecholamine-driven lipolysis and efficient fatty-acid transport into mitochondria support MFO, whereas increasing intensity elevates glycolytic flux and malonyl-CoA production, which inhibits carnitine palmitoyltransferase-1 (CPT-1) and limits long-chain fatty acid entry [8,9]. Consistent with this, Chatzinikolaou et al. [10] and Petridou et al. [11] reported that the capacity to sustain high rates of lipolysis and fatty acid oxidation is closely associated with improved metabolic flexibility and exercise adaptation. In both lean and obese men, resistance and endurance exercise markedly increase adipose tissue triacylglycerol lipase activity [10,12]. However, in obesity, this response appears delayed or prolonged and is accompanied by higher respiratory exchange ratio (RER) values, suggesting reduced utilization of mobilized fatty acids at the muscle level [10,11,12]. Such kinetics may partly explain inter-individual variability in Fatmax despite comparable lipolytic stimulation.

In obesity and insulin resistance, elevated basal insulin concentrations and blunted catecholamine responses suppress adipose tissue lipolysis and reduce plasma free fatty acid availability, while mitochondrial dysfunction and altered substrate delivery further constrain oxidation capacity [13,49]. Consequently, the intensity eliciting Fatmax often shifts toward lower workloads, and maximal rates of fat oxidation decline compared with metabolically healthy individuals [4]. Comparative data across populations further contextualize these findings. In endurance-trained athletes, Fatmax typically occurs at higher relative intensities (≈60–70% VO_2_max) with MFO values exceeding 0.6–0.8 g·min^−1^, reflecting superior mitochondrial oxidative capacity and enhanced lipid transport [6,21]. In contrast, untrained normal-weight adults generally exhibit Fatmax around 50–60% VO_2_max with MFO values of 0.3–0.4 g·min^−1^, whereas individuals with obesity or metabolic syndrome display lower values, often between 40–50% VO_2_max and 0.2–0.3 g·min^−1^ [48]. Aging also contributes to a downward shift in Fatmax and MFO, likely due to reduced mitochondrial volume and hormonal responsiveness [50]. These comparisons indicate that fitness level and metabolic health, more than body composition alone, are the primary drivers of inter-individual differences in fat oxidation capacity. Taken together, the main physiological determinants of Fatmax include hormonal milieu (insulin, catecholamines), substrate availability and transport, mitochondrial oxidative capacity, and prior training status [4,8,9,21].

### 4.3. Exercise and Training Interventions

Exercise interventions consistently improved Fatmax and MFO, often within a matter of weeks, emphasizing the plasticity of fat oxidation capacity [30,35,41]. These adaptations occurred across a spectrum of training modalities, including moderate endurance, HIIT, and Fatmax-targeted programs. Notably, exercise interventions specifically designed around Fatmax appeared to confer superior improvements in insulin sensitivity, suggesting that protocol intensity tailored to optimize fat metabolism may yield distinct clinical benefits [32,41].

These findings are consistent with broader literature showing that both moderate and high-intensity training improve metabolic outcomes, but they also highlight the unique potential of Fatmax-based prescriptions for dysmetabolic populations [30,41]. Importantly, mechanistic work indicates that training-induced increases in whole-body MFO may primarily reflect greater reliance on plasma free fatty acids rather than intramuscular lipid stores [40]. This observation aligns with metabolic tracer studies showing a preference for circulating substrates in obesity, raising important questions about the regulation of intramuscular lipid utilization in this population [41].

### 4.4. Nutritional and Supplementation Factors

Only limited evidence was available on nutritional or supplementation strategies. The lack of effect of dihydrocapsiate supplementation underscores the difficulty of pharmacological or dietary aids in substantially enhancing exercise fat oxidation, particularly compared with the robust effects of training [45]. Broader literature suggests that acute dietary manipulations (e.g., low-carbohydrate availability) can shift substrate utilization, but the long-term interaction between diet, supplementation, and Fatmax in populations with obesity remains poorly characterized.

### 4.5. Synthesis and Implications

Taken together, the evidence indicates that Fatmax is a physiologically meaningful and modifiable marker, particularly sensitive to training adaptations [32,35,41]. However, methodological inconsistencies, intra-individual variability, and limited standardization restrict its application as a clinical tool at present [24,25]. Nevertheless, the consistent improvements in MFO and shifts in Fatmax with training suggest that individualized prescriptions targeting this domain may enhance metabolic health beyond what is achieved with untailored exercise [32,41]. This is particularly relevant for obese and metabolically impaired populations, in whom optimizing fat oxidation could play a role in improving insulin sensitivity and reducing cardiometabolic risk [34,44].

### 4.6. Future Research Directions and Proposed Guidelines

To maximize the scientific and clinical utility of MFO and Fatmax assessment in individuals with obesity, future research efforts must focus on two integrated priorities: protocol standardization and mechanistic exploration.

#### 4.6.1. Establishing Standardized MFO Assessment Guidelines

The critical first step is to establish consensus on standardized Fatmax protocols by defining key methodological parameters to minimize methodological variability. Based on the synthesized evidence, we propose the following guidelines for cycle ergometer testing in obese populations:

##### Pre-Test Standardization

Pre-test conditions should be strictly controlled and standardized regarding:

- Nutritional State: Testing must be performed after an overnight fast (10–12 h).
- Physical Activity and Lifestyle: Standardizing dietary intake, caffeine consumption, and physical activity during the preceding 24–48 h is mandatory to control for acute metabolic influences.
- Circadian Effects: All testing should be conducted at a consistent time of day to account for circadian metabolic variations.

##### Protocol Design

Graded incremental tests should employ:

- Starting Workload: A low starting intensity of 20–30 W to avoid premature fatigue.
- Workload Increments: Small increments of 10–15 W to ensure multiple data points are captured in the low-to-moderate intensity range, where FATmax typically occurs in this population.
- Stage Duration: 4 min stages are preferred over the traditional 3 min stages to ensure near–steady-state gas exchange is achieved. The evidence suggests that 3 min stages may underestimate lipid oxidation fluxes compared to longer steady-state periods.

##### Data Analysis

Gas-exchange data should be averaged over the final 2 min of each stage, and substrate oxidation calculated using the Frayn equation. MFO should be identified as the highest measured value or via third-order polynomial fitting, with Fatmax expressed as both %HRpeak and %VO_2_peak, giving preference to heart-rate-based indices due to their reported lower intra-individual variability (CV ≈ 8.8%).

Implementing these standardized parameters would reduce methodological heterogeneity and improve the reliability and comparability of Fatmax outcomes across studies in individuals with obesity.

#### 4.6.2. Addressing Variability and Translational Value

In parallel, greater attention is required to clarify the determinants of inter-individual variability, including the relative roles of fitness level, adiposity, metabolic status, and hormonal factors in shaping Fatmax and MFO responses.

- Mechanistic Integration: Future studies should combine whole-body assessments with muscle-level and metabolic analyses (metabolic phenotyping), utilizing techniques like tracer methodology and muscle biopsy, thereby elucidating whether improvements in MFO reflect enhanced substrate delivery, mitochondrial adaptations, or changes in lipid partitioning.
- Clinical Expansion and Diversity: Research should address pediatric and sex-specific responses, using larger and more representative cohorts to explore how age, pubertal status, and sex hormones influence substrate utilization. Furthermore, expanding the clinical applications of Fatmax testing to diverse patient populations, such as those with cardiovascular disease or non-alcoholic fatty liver disease, may also provide valuable diagnostic and prognostic insights.
- Underexplored Interventions: Nutritional interventions remain underexplored, and further investigations into dietary strategies (e.g., low-carbohydrate availability), supplementation, and nutrient–exercise interactions are warranted.
- Long-Term Outcomes: Finally, long-term randomized controlled trials are required to determine whether Fatmax-targeted exercise interventions can deliver sustained benefits in weight management, metabolic health, and the prevention of chronic cardiometabolic disease

## 5. Conclusions

This review underscores the necessity of adapted exercise protocols for individuals with overweight and obesity in order to facilitate effective Fatmax assessment, with lower workloads, longer stages, and strict control of testing conditions. Fatmax typically occurs at lower relative intensities in sedentary individuals and those with obesity. However, cardiorespiratory fitness appears to be a stronger determinant than adiposity per se. Additional factors such as sex, developmental stage, and metabolic phenotype further modulate fat oxidation responses. Exercise interventions, particularly those targeting Fatmax, can rapidly enhance fat oxidation capacity and improve metabolic health, although mechanistic understanding remains incomplete. The adoption of standardized methodologies and the inclusion of larger, more diverse studies are needed to clarify variability and optimize the application of Fatmax in both research and clinical practice.

## Figures and Tables

**Table 1:** Employed studies.

Author	Subjects (n)	Age (yrs)	BMI (kg/m^2^)	GXT (Cycle Ergometer)	Fasting Condition	VO_2_max (mL/kg/min)	MFO (g/min)	FATmax (%VO_2_peak)	Fitness Status
Alkahtani et al. (2014) [29]	n = 12 M	29 ± 4.1	29.1 ± 2.4	Discontinuous graded cycling test: start 35 W, 4 min work + 4 min rest, +17.5 W each stage until RER ≥ 1.0. MFO from VO_2_/VCO_2_ (Frayn equation). Fatmax = workload at MFO. VO_2_max test followed after rest: start 2 stages lower than RER 1.0-point, +17.5 W/min to exhaustion	12 h	31.8 ± 5.5	0.14 ± 0.08	34 ± 0.02	Sedentary overweight/obese men
Alkahtani SA et al. (2013) [30]	n = 10 M	29 ± 3.7	30.7 ± 3.4	Discontinuous graded cycling test (Fatmax protocol): 4 min work at 35 W + 4 min rest, +17.5 W each stage until RER ≥ 1.0. MFO from VO_2_/VCO_2_ (Frayn eq.). Fatmax = workload at MFO. After rest, VO_2_max test: start two stages below RER = 1.0 workload, +17.5 W/min until exhaustion	12 h	28.7 ± 3.4	0.13 ± 0.07	N/D	Sedentary overweight/obese men
Ara I et al. (2011) [31]	n = 30 M, O (10), PO (10), C (10)	O: 30.4 ± 2.3 PO: 31.5 ± 1.6 C: 31.2 ± 1.5	O: 33.8 ± 1.0 PO: 26.6 ± 0.7 C: 26.6 ± 0.6	Two graded exercise tests (lower body cycling and upper body arm cranking). Leg test: start at low workload, +increments every 3 min until RER > 1.0. Arm test: start 20 W, +15 W every 3 min until RER > 1.0. VO_2_ and VCO_2_ measured continuously. MFO and Fatmax from polynomial fit (Frayn equation)	Overnight fast	O: 3.4 ± 0.2 PO: 3.7 ± 0.1 C: 3.6 ± 0.1	O: 0.40 ± 0.04 PO: 0.39 ± 0.02 C: 0.26± 0.02	O: 47.4 ± 1.5 PO: 45.3 ± 2.6 C: 38.3± 1.8	Sedentary to moderately active men, post obese, obese, and normal-weight controls
Besnier F et al. (2015) [32]	n = 136, Ov/Ob Women (Ov/Ob W)	Ov/Ob W: 30.1 ± 5.6	Ov/Ob W: 33.1 ± 3.5	Metabolic cycling test: stages at 20%, 30%, 40%, 50%, 60% of MAP (5–6 min each); Indirect calorimetry; MFO determined from VO_2_ and VCO_2_; VO_2_peak test: start at 20 W, +15 W/min until exhaustion	Overnight fast	Ov/Ob W: 42.5 ± 6.3	Ov/Ob W: 0.15 ± 0.04	Ov/Ob W: 45.7 ± 8.3	Young sedentary overweight/obese women
Brandou F et al. (2005) [33]	n = 15 B, 7 (LI) 8 (HI)	LI: 11.8 ± 0.5 HI: 12.8 ± 0.5	LI: 29.5 ± 1.7 HI: 30.1 ± 1.7	Graded cycling test: 5 × 6 min steady-state workloads at 20, 30, 40, 50, 60% of Wmax(th) (determined from theoretical VO_2_max). VO_2_ and VCO_2_ measured in the last 3 min of each stage. Fat oxidation calculated via non-protein RQ technique; Lipox max (MFO intensity) identified from smoothed curve for training prescription. LI group trained at Lipox max, HI group at Lipox max + 40%	12 h	N/D	N/D	LI: 50.8% ± 2.6% Wmaxth HI: 60.8% ± 5.3% Wmaxth	Obese boys on hypocaloric diet. pre-, pubertal, and post-pubertal stages
Cancino Ramírez J et al. (2018) [34]	n = 60, No-IR (n = 22), IR (n = 38)	No-IR: 35.4 ± 10.2 IR: 30.6 ± 8.5	No-IR: 29.4 (28.1–31.8) IR: 32.8 (30.6–35.9)	Incremental cycle ergometer test with gas analysis. 3 min rest, 3 min warm-up at 20% Wt, then 6 min stages at 30, 40, 50, 60% Wt until RER ≥ 1.0, followed by 1 min stages (+10% Wt) until exhaustion. VO_2_ and VCO_2_ analyzed. MFO calculated via Frayn equation	6 h	No-IR: 24.3 ± 3.1 IR: 23.9 ± 3.2	No-IR: 0.76 ± 0.66 IR: 0.54 ± 0.5	No-IR: 46.1 ± 9.0 IR: 45.3 ± 8.5	Sedentary overweight/obese women, grouped by insulin resistance status
Cao J et al. (2022) [35]	n = 56, EXP (31), CON (25)	EXP: 41.2 ± 8.9 CON: 40.1 ± 6.9	EXP: 26.4 ± 4.1 CON: 25.2 ± 2.0	Graded cycling test: start at 50 W, +20 W every 3 min until exhaustion; cadence ≥60 rpm; Indirect calorimetry; MFO from VO_2_ and VCO_2_; Fatmax determined as intensity at MFO.	12 h	EXP: 29.1 ± 5.1 CON: 30.3 ± 5.5	EXP: 0.24 ± 0.08 CON: 0.25 ± 0.08	EXP: 50 ± 6 CON: 49 ± 7	Sedentary
Crisp NA et al. (2012) [36]	n = 10, Overweight Boys (Ov B)	Ov B: 10.3 ± 1.9	Ov B: 25.0 ± 4.0	Two protocols: (1) GRAD: 3 min stages at 35, 40, 45, 50, 55, 60, 65% VO_2_peak; (2) PROL: 30 min steady-state cycling at 40, 45, 50, 55, 60% VO_2_peak	Overnight fast	Ov B: 34.0 ± 7.2	GRAD: 0.303 ± 0.08 PROL: 0.328 ± 0.06	GRAD: 53 ± 10 PROL: 53 ± 10	Overweight boys, sedentary
Croci I et al. (2014) [37]	n = 24, HiFat (12M), LoFat (12M)	HiFat: 37 ± 10 LoFat: 38 ± 9	HiFat: 29.3 ± 3.6 LoFat: 24.0 ± 2.5	Graded cycling test (Excalibur Sport): 15 min seated baseline, 5 min warm-up at 60 W, then start at 60 W, +30 W every 4 min until exhaustion. Indirect calorimetry, Fat oxidation from Frayn equation. Fatmax and MFO determined using the SIN model from multiple steady-state stages	12 h	HiFat: 39.0 ± 5.5 LoFat: 41.4 ± 7.6	HiFat: 0.42 ± 0.16 LoFat: 0.38 ± 0.19	HiFat: 45.4 ± 7.2 LoFat: 46.7 ± 8.6	Recreationally
Dandanell S et al. (2017) [24]	n = 24, Ob (8M/8F) Non Ob (5M/3F)	Ob: 28 (26–29) Non Ob: 32 (30–34)	Ob: 36 (35–38) Non Ob: 23 (22–24)	Graded cycling: 5 min rest, 6 min warm-up at 30 W, then +20 W (women)/+25 W (men) every 3 min until RER > 1.0. Compared with Short Continuous Exercise (SCE): 10 min rest, then 10 min at 35%, 50%, 65% VO_2_max (randomized order)	12 h	Ob: 29.8 (28.2–31.4) Non Ob: 45.2 (44–47)	Ob: 0.33 (0.29–0.37) Non Ob: 0.31 (0.25–0.38)	Ob: 42.1 (38.5–45.7) Non Ob: 42.3 (40.1–44.4)	Ob: Non-trained, Non Ob: moderately trained
Dumortier M et al. (2003) [38]	n = 39, T (7M/21F), C (4M/7F)	T: 52.0 ± 2.4 C: 52.7 ± 3.4	T: 32 ± 1.7 C: 33.9 ± 2.6	Submaximal incremental cycling test: 3 min warm-up at 20% theoretical Wmax, then 4 × 6 min steady-state stages at 30%, 40%, 50%, 60% Wmax. Gas exchange measured in last 3 min of each stage	12 h	T: 17.21 ± 1.17 C: 19.95 ± 3.27	N/D	N/D	Overweight/obese sedentary insulin-resistant patients with metabolic syndrome
Guiraudou M et al. (2015) [39]	n = 47, Ob	50.9 ± 2.1	35.1 ± 0.9	Graded cycling test: 5 × 6 min steady-state workloads theoretically set at 20, 30, 40, 50, and 60% of Pmax at the crossover point (70% carbohydrates and 30% lipids)	12 h	N/D	N/D	N/D	Sedentary obese patients
Ipavec-Levasseur S et al. (2015) [40]	n = 23, Ob (18M), Non Ob (5M)	Ob: 44 ± 7 Non Ob: 44 ± 9	Ob: 36.8 ± 3.6 Non Ob: 24.3 ± 2.1	Graded cycling test: 4 min stages, +0.3–0.4 kg resistance each stage until RER > 1.0; Fat oxidation calculated via Frayn equation. Fatmax determined graphically. 1-h steady-state cycling at Fatmax performed pre/post lifestyle intervention	12 h	Ob: 25.6 ± 5.7 Non Ob: 57.9 ± 2.7	Ob: 0.33 ± 0.19 Non Ob: 0.68 ± 0.08	Ob: 61.9 ± 12.4 Non Ob: 59.8 ± 8.4	Ob: Non-trained, Non Ob: recreationally trained men
Chrzanowski-Smith OJ et al. (2020) [25]	n = 99, H (30M/40F) OV (18M/9F) OB (2M/0F)	35 ± 11	H: 21.9 ± 2.2 OV: 26.6 ± 4.9 OB: 31.8 ± 2.2	Incremental graded cycling test. First 7 stages: 4 min each, +25 W (except 30 → 40 W stage = +10 W). From stage 8 onwards: 2 min stages, +50 W until exhaustion. Expired gas collected via a Douglas bag. VO_2_/VCO_2_ measured. Fat oxidation from the Frayn equation. Fatmax = intensity at PFO (g/min)	10–12 h overnight fast	H: 21.9 ± 2.2 OV: 26.6 ± 4.9 OB: 31.8 ± 2.2	42.2 ± 10.3	39 ± 10	Healthy adults, wide range of CRF, sedentary to very active
Lanzi S et al. (2015) [41]	n = 19, Gfatmax (10M), Ghiit (9M)	Gfatmax: 38.1 ± 2.3 Ghiit: 34.9 ± 3.4	Gfatmax: 40.9 ± 1.1 Ghiit: 43.1 ± 1.0	Maximal incremental cycling test (IncrP1: 5 min stages at +10% PPO_ramp until RER 1.0. IncrP2: +15 W/min until exhaustion). GFatmax: 40–50 min continuous cycling at individual Fatmax. GHIIT: 10 × 60 s at ~90% HRmax, 60 s recovery, total 30 min. 8 supervised sessions in 2 weeks	12 h	Gfatmax: 23.1 ± 1.2 Ghiit: 24.1 ± 1.2	Gfatmax: 0.43 ± 0.04 Ghiit: 0.45 ± 0.01	Gfatmax: 48.8 ± 2.9 Ghiit: 52.3 ± 2.7	Sedentary men with severe obesity (Class II–III), in a lifestyle intervention program
Lanzi S et al. (2014) [42]	n = 32, L (16M) O (16M)	L: 33.1 ± 1.6 O: 34.5 ± 2.1	L: 22.9 ± 0.3 O: 39.0 ± 1.4	Submaximal incremental cycling test: 10 min warm-up at 20% PPO, then +7.5% PPO every 6 min until 65% PPO or RER = 1.0; Indirect calorimetry; Fat oxidation via Frayn equation; SIN model used to determine MFO and Fatmax	12 h	L: 41.8 ± 1.8 O: 25.2 ± 0.9	L: 56.8 ± 1.7 O: 47.2 ± 2.6	L: 56.8 ± 1.7 O: 47.2 ± 2.6	Sedentary, lean and obese men
Lazzer S et al. (2007) [2]	n = 60, Ob (15B/15G) Non Ob (15B/15G)	14–16	Ob B: 33.8 ± 5.3 Ob G: 33.8 ± 4. Non Ob B: 21.9 ± 5.1 Non Ob G: 23.5 ± 5.1	Two graded cycling tests on an electromagnetically braked ergometer: (1) VO_2_peak test: start at 30 W, +30 W every 2 min until HR = 180 bpm. (2) MFO/Fatmax test: 5 min warm-up at 10% VO_2_max, then 5 min steady-state stages at 30%, 40%, 50%, and 60% VO_2_max at 60 rpm. VO_2_ and VCO_2_ measured in the last minute of each stage	12 h	Ob B: 52.5 ± 1.6 Ob G: 47.8 ± 1.6 Non Ob B: 54.2 ± 1.9 Non Ob G: 50.5 ± 1.7	B: 0.32 ± 0.02 G: 0.25 ± 0.02	Ob B: 40 ± 3 Ob G: 38 ± 3. Non Ob B: 45 ± 3 Non Ob G: 42 ± 5	Sedentary, severely obese and sedentary nonobese adolescents
Makni E et al. (2012) [43]	n = 131 children, B (68) G (63)	B: 13.4 ± 1.1 G: 13.8 ± 0.9	B: 29.2 ± 3.3 G: 31.0 ± 4.0	Submaximal incremental cycling test: 3 min rest, then 6 min stages at 20%, 30%, 40%, 50%, 60% of predicted Wmax. VO_2_ and VCO_2_ measured during final 2 min of each stage. Lipid oxidation calculated via equation. Fatmax defined as the stage with the highest fat oxidation. Also performed 6 min walking test (6MWT) to predict Fatmax.	N/D	B: 1.97 ± 0.08 L/min G: 1.99 ± 0.19 L/min	B: 0.12 ± 0.01 G: 31.0 ± 4.0	B: 75.7 ± 12.1 W G: 76.2 ± 10.0 W	Sedentary obese children
Mogensen M et al. (2009) [44]	n = 23, T2D (12), C (11)	T2D: 52.5 ± 1.4 C: 53.6 ± 1.8	T2D: 33.5 ± 1.0 C: 33.2 ± 0.8	Cycling test: warm-up, then start at 30 W with +30 W every 4 min until RER = 1.0	12 h	T2D: 26.7 ± 1.0 C: 29.0 ± 1.7	T2D: 0.28 ± 0.02 C: 0.32 ± 0.03	T2D: 39.7 ± 3.9 C: 43.7 ± 4.1	Obese sedentary men with and without type 2 diabetes
Osuna-Prieto FJ et al. (2022) [45]	n = 24 M	40.2 ± 9.2	31.6 ± 4.5	Graded cycling (start 20 W, +20 W every 3 min until RER ≥ 1.0) to determine MFO and Fatmax	5–6 h	30 ± 6	0.24 ± 0.09	33 ± 7	Sedentary overweight/obese men
Ounis OB et al. (2009) [46]	n = 54 adolescents, B (27) G (27)	13.2 ± 0.8	30.3 ± 4.0	Graded cycling test: a 3 min rest period was followed by a 5-stage progressive submaximal exercise test (20, 30, 40, 50, and 60% of Wmax) with 6 min of exercise at each work rate and a 5 min active recovery period at 20% of Wmax.	12 h	N/D	0.19	N/D	Obese adolescents without a medical history
Tsujimoto et al. (2012) [15]	n = 15, Obese Men (Ob M)	Ob M: 53.5 ± 6.9	Ob M: 27.8 ± 1.7	Graded cycling test: start at 15 W, +15 W every 4 min until RER > 1.00; Expired gas collection via indirect calorimetry	Overnight fast (≥8 h)	Ob M: 27.7	Ob M: 0.22 ± 0.023	Ob M: 34.8 ± 1.6	Middle-aged obese men, untrained
Zunquin G et al. (2009) [47]	n = 46, PrePub (16), Pub (16), PostPub (14)	PrePub: 9.7 ± 0.7 Pub: 11.9 ± 0.5 PostPub: 14.6 ± 0.6	PrePub: 22.7 ± 2.5 Pub: 27.2 ± 3.4 PostPub: 29.3 ± 2.6	Graded cycling test: 30 W start, +20 W every 3.5 min, cadence ~60 rpm, Indirect calorimetry, Fat oxidation from VO_2_ and VCO_2_, Warm-up 5 min at 0 W	Standard breakfast at 07:00, test 2 h later (non-fasted overnight)	PrePub: 33.84 ± 4.70 Pub: 29.49 ± 5.34 PostPub: 28.90 ± 5.01	PrePub: 0.44 Pub: 0.41 PostPub: 0.34	PrePub: 49.47 ± 1.62 Pub: 47.43 ± 1.26 PostPub: 45.00 ± 0.87	Obese boys at different pubertal stages

## Data Availability

The original contributions presented in this study are included in the article. Further inquiries can be directed to the corresponding authors.

## Supplementary Materials

The following supporting information can be downloaded at https://www.mdpi.com/article/10.3390/diseases14010004/s1 Table S1: Preferred Reporting Items for Systematic reviews and Meta-Analyses extension for Scoping Reviews (PRISMA-ScR) Checklist.
